# Large Animal Emergency Relief Services—A Model for University Engagement With Private Practitioners and Development of Practice Readiness for Veterinary Students

**DOI:** 10.3389/fvets.2020.00403

**Published:** 2020-07-17

**Authors:** Meggan T. Graves, David E. Anderson, Robert C. DeNovo

**Affiliations:** ^1^Department of Large Animal Clinical Sciences, College of Veterinary Medicine, University of Tennessee, Knoxville, Knoxville, TN, United States; ^2^Administration and Hospital Programs, College of Veterinary Medicine, University of Tennessee, Knoxville, Knoxville, TN, United States

**Keywords:** large animal, emergency, relief practice, equine, food animal, after-hours

## Abstract

Large animal teaching hospitals often struggle to maintain consistent teaching caseloads, which are affected by seasonal variations, economic pressures, increased abilities of local large animal practices to hospitalize large animals, and client intolerance for the operational needs of an academic mission. Non-academic large animal practices enjoy a more consistent caseload but suffer from a lack of emergency relief and a limited ability to share emergency duties, which may have adverse effects on work-life balance. An academic, on-farm, large animal emergency relief service can combine multiple clinics' emergency services to increase overall caseload and the probability of consistent teaching exposure for veterinary students. In late November of 2013, the Large Animal Teaching Hospital at the University of Tennessee, College of Veterinary Medicine adopted a business model to provide a large animal emergency relief service to area practitioners; enhance student learning via increased emergency caseload; and advance the academic mission to develop practice-ready graduates. Providing this service contributes to the well-being of area practitioners and enriches student learning through increased caseload.

## Introduction

Large animal teaching hospitals often struggle to maintain consistent teaching caseloads. Teaching hospital caseload may be affected by seasonal variations, economic pressures, increased abilities of local large animal practices to hospitalize large animals, and client intolerance for the operational needs of an academic mission. Non-academic large animal practices enjoy a more consistent caseload but suffer from a lack of emergency relief and a limited ability to share emergency duties, which may have adverse effects on work-life balance (1).{Ramey, 2012, Equine ambulatory practice: challenges and opportunities} In a study of rural practice economics, 68% of private mixed animal practitioners reported that they spent > 10% of their time responding to unscheduled sick animal calls and that these occurred with 83% of their clients (2). The contemporary model used by small animal practices to provide emergency services is to refer their clients to an emergency clinic, which returns the case to the private practice on the next regular workday (3). This model may benefit partially because of population density of small animal owners, but also because owners can usually easily transport the patient to the facility. Large animal emergency services, on the other hand, are expected to have smaller caseloads because of population dispersion of the animals, challenges associated with transporting large animals, cost of care and, in many cases, limited expected economic return to the owner. Therein exists a niche for on-farm, large animal emergency relief services. In a survey of rural practicing veterinarians and veterinary students regarding their confidence in performing practice procedures, emergency cases such as colic, dystocia, and death outbreaks were rated low for practice readiness (4). Perhaps not surprisingly, new graduates rated emergency duty workload highest, in addition to inadequate personal time, in the decision to leave rural veterinary practice (5). If centrally located, the emergency caseloads for multiple clinics could support emergency relief clinicians. Additionally, combining multiple clinics' emergency services increases caseload and the probability of consistent emergency case teaching exposure for veterinary students. This case based experience is expected to improve the confidence, practice readiness, and retention of new graduates entering rural practice. In late November of 2013, the Large Animal Teaching Hospital at the UTCVM adopted a business model to provide a large animal emergency relief service to area practitioners, enhance student learning via increased emergency caseload, and advance the academic mission to develop practice-ready graduates.

## Business Model

The sustainability of an emergency relief service is predicated on the participation of the regional large animal practitioners so that the caseload, cumulatively, is sufficient for the relief service to be self-supporting. Therefore, the first step in building the business model was for the emergency clinician to engage local large animal practitioners with a practice radius of 40-miles (1-h drive) from the teaching hospital. Communication was via personalized letter (Supplement 1), telephone, e-mail, and/or personal contact to make the practitioners aware of the availability of the new service, as well as, the experience and competency of the emergency clinician. Personal contact is preferred to allow the local practitioner an opportunity to meet the emergency clinician, explain their practice dynamic, and establish a relationship with the clinician. A critical component of the service was assuring potential collaborating veterinarians that no preventative or routine care services would be provided by the emergency service and that all cases would return to the practice at the start of the next business day unless referral to a surgical facility prevented such. Vital to the relationship was also awareness that the emergency clinician would be employed by the UTCVM only for after-hours emergency services, with no connection to routine, primary-care services, or regular workday activities. This allows the participating practitioner to be confident that competition for routine services will be avoided. Additional advantages for the practitioner include direct billing of emergency clients, which off-loads workload from the practice related to business office including delays or failure of payment.

After establishing a potential client base, an emergency calendar schedule with available coverage days (i.e., Monday to Thursday after 5 p.m., Friday 5 p.m. to Monday 8 a.m., and holidays) was generated consisting of ~70% coverage. This schedule was circulated to participating veterinary practices, and each practice then decided on a consistent method of transferring or forwarding emergency calls to the emergency clinician. Practices could elect to forward their emergency calls directly to the emergency clinician, have their answering service contact the emergency clinician, or simply leave the emergency clinician's contact information on their messaging service. Some veterinarians chose to use the relief service selectively by fielding their own calls and selecting which cases they see themselves and which ones they forward to the emergency clinician. This flexibility allows private practitioners to adapt this service to best fit their practice or individual needs. By maintaining the practice's control over the use of the service, practitioner trust, confidence, and interdependence can be increased over time.

Following patient care, the emergency relief service clinician notifies the primary veterinarian of the patient that was seen on emergency and may also communicate any client communication or follow-up patient care that may be required. All subsequent patient needs will be met by the primary veterinarian, ensuring the non-competitive nature of the service, unless otherwise directed by the private practitioner.

The large animal emergency relief service at the UTCVM is provided at no cost to the local practitioner. Any revenues generated come directly from the clients served at the time of the emergency. Clients pay the university directly with no monies exchanged through the participating practice. In the event that a practice wanted to maintain continuity of its fee structure, the relief service could bill the practice and then the practice bill the client. Within the overall business of a practice, emergency services may be at higher risk of increasing delinquent accounts receivable. The relief service provides a buffer between the practice and these clients and this is seen as an added value by private practices.

## Outcomes

### Number of Participating Practices

Practices were given autonomy regarding the level of engagement of the service. In the first 3 years of the Large Animal Emergency Relief Service, 4 practices consistently utilized the relief service for available dates. Solo practitioners comprised 3 out of 4 of those practices. Six additional practices used the service intermittently and at their convenience. Practices that did not consistently utilize the service expressed reliance on revenues generated from emergency services or a lower emergency caseload burden shared among associates. All but one of the consistent practices are strictly ambulatory with a variety of large animal species treated. One participating practice has a full service hospital; cases of clients of this practice requiring hospitalization or surgery are admitted to the practice by the emergency clinician. The case is then transferred to an associate of the practice.

### Changes in Monthly Caseload

**Figure 1:**
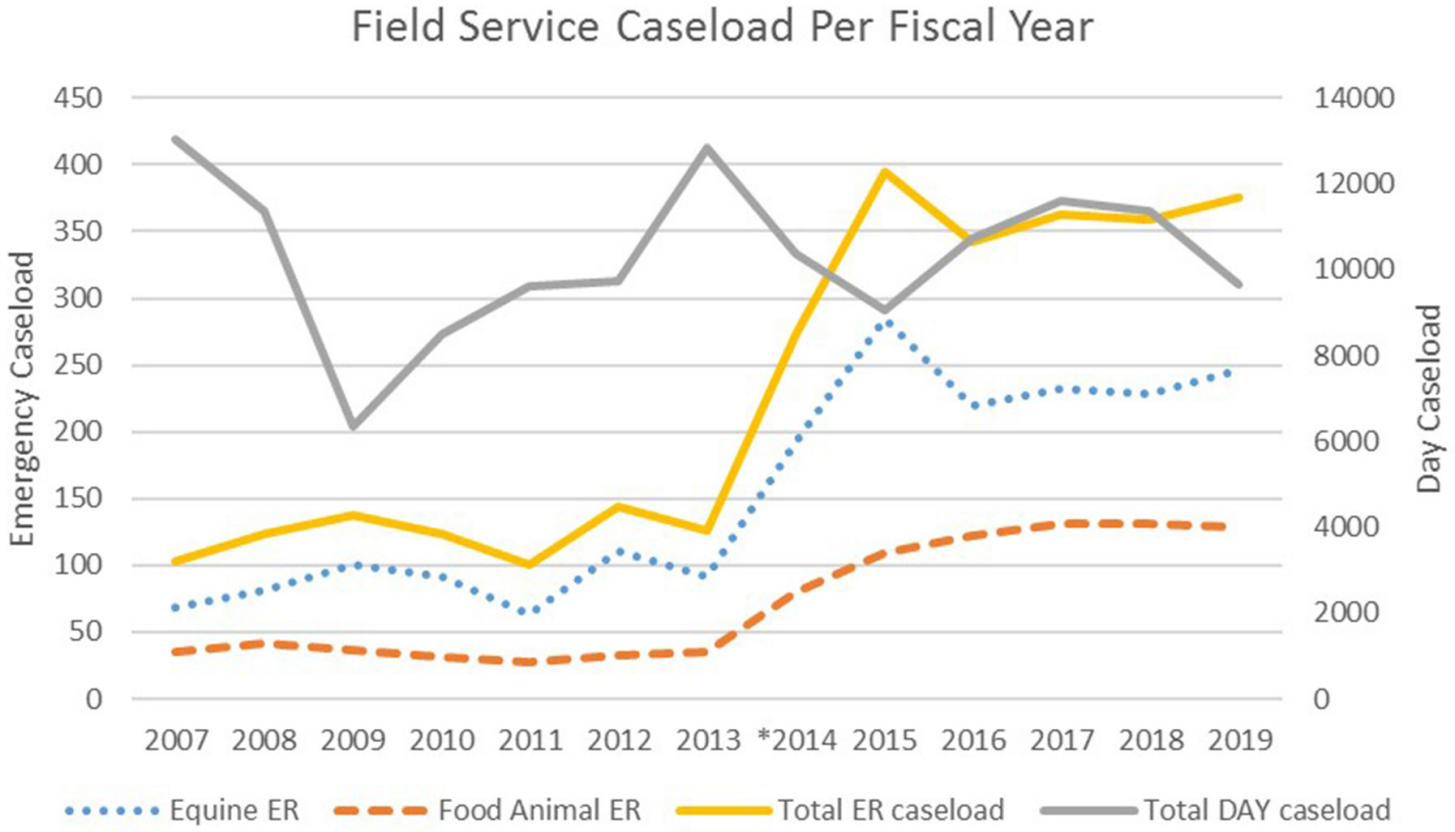
Field Service emergency caseload pre- and post- relief service establishment in November 2013. *formation of emergency service.

**Figure 2:**
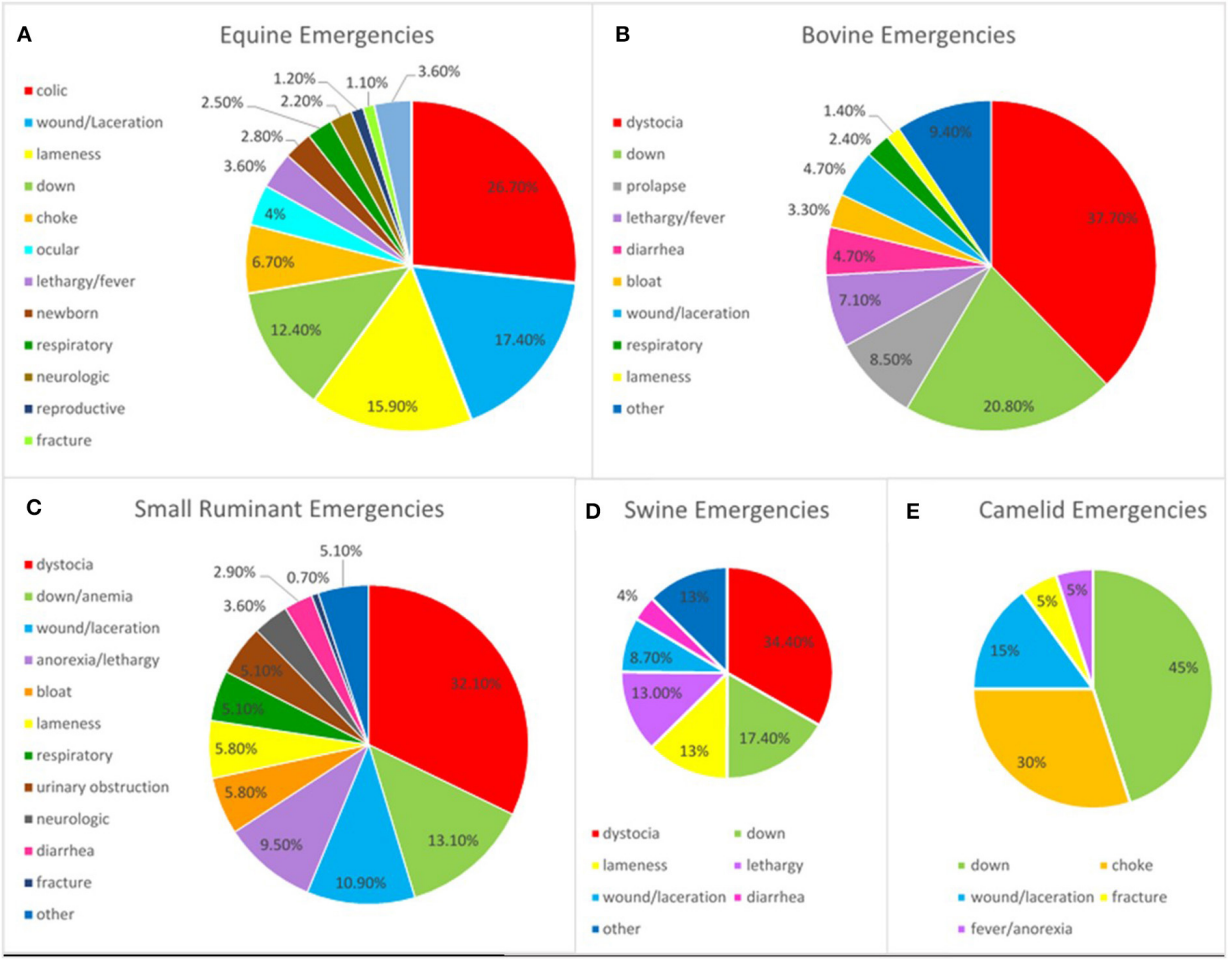
Emergencies attended after-hours from 2015 to 2019 based on presenting complaint for equine **(A)**, bovine **(B)**, small ruminant **(C)**, swine **(D)**, and camelid **(E)** patients.

In the decade prior to beginning this collaborative service, after-hours emergency caseload for field services at the UTCVM had seen a period of general decline. Although the daytime field service caseload was thriving, the evening service lacked a strong caseload for teaching. An average of 124 cases were attended annually. Since the development of the emergency service in November 2013, the annual field-based caseload increased to ~400 cases in 2015 (Figure 1). With the implementation of this business model, the number of both food animal and equine emergencies has increased. Approximately 65% of emergencies are equine, and 35% are livestock, making it important that the emergency clinician be comfortable seeing a variety of large animal cases. Anything considered a concern to the owner warrants an emergency call. Emergency cases are typical for the southeastern region with a mix of pleasure and competition equids, cow-calf operations, and backyard sheep and goat herds predominating. The most common equine emergencies attended are colics, wounds or lacerations, lamenesses, and the recumbent horse (Figure 2A). Common presenting complaints for bovine emergencies were dystocia, down for variety of reasons, or other reproductive issues such as uterine or vaginal prolapse (Figure 2B). The small ruminant caseload is similar to bovine, with dystocia most prevalent (Figure 2C). Swine and Camelid emergencies only account for roughly 3% of all emergencies attended and are summarized in Figures 2D,E.

### Changes in House Officer Training

Before implementation of the large animal emergency relief service, house officers (predominately interns) routinely attended emergencies on their own in the field, and a senior clinician acted in a back-up role via telephone consultation only. This system did not allow for direct supervision or hands-on oversight. In addition, clients frequently complained about their impression that the emergency clinician was not willing or responsive to their needs. This was interpreted as a consequence of fatigue much like rural practitioner experience. House officers could relay case information to their senior clinician, but the clinician was unable to interpret the accuracy of the findings. Therefore, any misinterpreted history or clinical finding by the house officer could not be used by the senior clinician to assist in proper diagnosis or treatment. With the new business model, house officers are given the direct mentorship by a clinician experienced in emergency services, client communication, triage, referral skills, and billing. This allows the emergency clinician to teach effective communication skills, as well as economic strategies from a practical viewpoint. Valuable triage skills can be learned through field-based emergency settings, which directly correlate to non-academic clinical practice and improve practice readiness.

### Changes in Student Training

Students on the field service rotation are required to complete emergency shifts in which they are on-call or available for 12-h periods for emergency response through the relief service. Students must be in the field service truck within 20 min of the call. Although this does increase response time for some emergencies, UTCVM being centrally located to many of the practices served makes the overall response time similar to solo practitioners. If an emergency is critical, the clinician may go directly to the call to avoid compromising patient care. With a single student covering a shift at a time, one-on-one teaching with the house officer and emergency clinician creates an environment for focused learning. This personal attention also affords students additional hands-on training as situations allow. In these settings, students are given client communication and patient care responsibilities with individual consideration to their development as future veterinarians.

### Relationships With Area Practices

Since introducing the UTCVM relief service, communication with local practices have increased due to case management. The emergency clinician routinely corresponds with participating practitioners for feedback or follow-up. The business model promotes interdependence such that the College of Veterinary Medicine offers a more direct benefit to the private practice and serves as a non-competitive asset. One practitioner that has benefited from this service is quoted as saying, “Having dependable emergency coverage allows me to plan on down time. I know the days aren't going to last into the night and the weekend really will come....that means a lot for my sanity!” (Gretchen Laws, DVM; personal communication). Comments such as these and the continued use of the emergency service is perceived evidence of practitioner satisfaction, although future efforts to survey participants would be valuable to understand the extent in which this service has improved quality of life or provided an added service to their practice.

## Benefits

As a result of diminishing state support for veterinary teaching hospitals, and teaching programs, self-supporting services are increasingly valuable. With the low overhead of field-based services, such a relief service can be self-sustaining from the revenues generated (1). The addition of this service required only the hire of a single faculty member since equipment and vehicles are shared with day faculty. An added teaching and revenue benefit is the opportunity for influx of referral cases to the Large Animal Hospital from field emergency cases. Since the implementation of this model, an average of 3.3 additional cases per month have been referred to the UTCVM for further diagnostics or treatment from the field-based emergency service.

Comparison of year 0 (prior to establishment of the service) to year 1 (first year of operation of the service), month-over-month revenue increased an average of 85% (Supplement 2). Increases based on species serviced were an average of 72% for equine service calls and 98% in farm animal service calls. Over the next 4 years, these revenue levels were maintained and fluctuated from the newly established baseline ranging from −9 to 15% per year. Fluctuations based on species service ranged from −16 to 21% for equine service calls and −1 to 24% for farm animal service calls. Interestingly, revenue fluctuations varied by species such that when revenue declined for equine, they rose for farm animal and vice versa. This helped to ensure stability of the service and demonstrates an advantage of a diversified service. Revenues from night and weekend field service emergency calls were not tracked separately from the day service prior to the establishment of the relief service; however, based on average emergency call invoices, annual revenue from field-based, after-hour emergencies were estimated to have increased by $83,000–166,000.

Data from national veterinary surveys highlight the need for providing emergency relief services to private large animal practitioners. The 2019 AAEP Annual Report states that ~40% of equine veterinarians are solo practitioners (6). The American Veterinary Medical Association reported in 2009 that solo practitioners represented 35% of food animal veterinarians (7). These facts, coupled with the more recent linkage of veterinarians with high rates of depression, suicide, and anxiety, make this relief service an opportunity for intervention, as well. Contributing work-related stressors include long work hours, high client expectations, and after-hours, on-call duties (5, 8). According to the 2019 AAEP Economic Report, nearly 64% of equine veterinarians report being on-call 100% of the time if in 2–3 doctor or solo equine practice. A major benefit of this model for area practitioners, especially solo practitioners, is the alleviation of a proportion of their after-hours emergency burden while, at the same time, upholding their clients' expectations for rapid, authoritative response to needs.

The emergency relief service provides an improved caseload for veterinary students and house officers accompanying the emergency clinician. Students are exposed to a greater number of diverse cases when collaborating with private practices. It is also a beneficial learning experience for students and house officers to be exposed to several different practice models.

New graduates are expected to demonstrate competence with handling routine emergencies such as simple lacerations, choke, colic, basic ophthalmic emergencies, and dystocias (9). When recruiters for food animal practices were asked to make recommendations to veterinary teaching hospitals about improving graduates for employment, 58% suggested increasing the food animal curriculum and practical experiences such as calving (10). Involvement in such emergency cases through this service model allows students and house officers to use communication skills and develop technical skills in patient care and client service.

## Challenges

A common conception held by private practitioners is that field-based services provided by a veterinary teaching hospital are in direct competition for clients. When practitioners are introduced to the emergency clinician, they are informed of the covenants of this service. Owners may request convenience services during an emergency call, but these requests are referred to the primary care veterinarian to perform. As a result, practitioners have been able to realize the mutually advantageous nature of such a service. A clinician solely dedicated to after-hour service is essential to ensure a lack of competition for routine day care and services.

Another challenge when establishing this type of service is that teaching hospitals are often viewed as being inefficient due to student participation. It is vital that house officer and student response times be enforced to ensure timeliness to emergency calls. Emergency services lend themselves, by the nature of the urgent care need, to a more efficient teaching exposure. Owners must be confident that patient care and competent emergency treatment will not be compromised because of teaching. For these reasons, an emergency relief service needs experienced personnel who are highly motivated to perform emergency services so that quality and willingness are conveyed to the client. The gravity of the situation will dictate the level of student involvement.

Although day services routinely have 5 or more students with them, this would potentiate issues of inefficiency or safety concerns if attempted with emergency services. Limiting participation to one student and one intern allows for individualized training and avoids owners who may not be used to clinical teaching from feeling overwhelmed by too many people involved. House officers are regularly assigned to Field Services as part of their training; therefore, other hospital services do not experience a void.

## Discussion

Work-life balance continues to increase in value among practitioners and new graduates, and more importance is being placed on time spent away from the workplace and raising families (5, 11). This change can be attributed in part to the changing demographics of graduating veterinarians. With women currently representing 80% of veterinary graduates, family-related needs may be of greater priority and concern (12, 13). In addition, there is a negative perception of the quality of life of large animal practitioners because of the high demands and long hours associated with large animal practice and emergency coverage. Many small animal practitioners rely on after-hours emergency clinics to extend their own excellent client service and patient care. This enables them to balance life and career without jeopardizing their veterinary practice. Large animal practitioners, however, traditionally have not experienced such opportunities. Students rated practice atmosphere and location as the most important factors in choosing their first rural practice job (5). However, as salary, emergency duty, and availability for personal time become higher priorities, these often result in these early career veterinarians leaving rural practices.

A 2012 AAEP survey reported that 41% of horse owners and trainers listed 24-h veterinary access as a top priority for choosing their veterinarian (14). Twenty-four hour availability is compounded by the reticence many solo practitioners have toward sharing emergency coverage because of the fear of potentially losing clients. Moreover, reports show that long work hours and after-hours work demands are the primary reason veterinarians leave mixed or large animal practice (15). Approximately half of new graduates who enter food animal practice change career paths within 5 years of graduation (11, 12). More than 60% of those individuals report that the substantial time demands of being on call is a primary contributing factor (16, 17). In an effort to combat this statistic and meet the needs of clients, the business model described herein was developed.

Many veterinary teaching hospitals are pursuing creative collaborations in order to meet the changing dynamics in veterinary education (18). The collaborative business model we adopted was designed to address one potential way that the UTCVM could help improve local practitioner quality of life, while also enhancing student learning and fulfilling the academic mission. Providing field-based, emergency relief services, in a non-competitive nature, allows the UTCVM to engage the practice community in a practical and life-enriching manner. Collaborating with the private sector also benefits veterinary students and house officers by affording additional case exposure and educational opportunities. Finally, the College benefits from a more interactive relationship with the rural practices which positively impacts the trust and collegiality needed in an increasingly complex environment.

## Summary

The UTCVM Department of Large Animal Clinical Sciences has created a large animal emergency relief service providing field-based emergency coverage to area practitioners with a non-competitive design model allowing for improved work-life balance to area veterinarians and greater emergency caseload for students. This service advances the academic mission by contributing to the development of practice-ready veterinarians.

## Data Availability Statement

The datasets generated for this study are available on request to the corresponding author.

## Author Contributions

MG developed the business model. DA and RD adopted the business model at the university. MG and DA collected the data and drafted the article. All authors contributed to the article and approved the submitted version.

## Conflict of Interest

The authors declare that the research was conducted in the absence of any commercial or financial relationships that could be construed as a potential conflict of interest.

## Supplementary Material

Supplement 1 Example personalized introductory letter to an area veterinarian announcing the new emergency service provided by the University of Tennessee College of Veterinary Medicine.

Supplement 2 Percentage change of revenue generated for fiscal year 2014 to 2019 from an after-hours, field-based, emergency relief service.

## Abbreviations

AAEP: American Association of Equine Practitioners
UTCVM: University of Tennessee, College of Veterinary Medicine.
