# Supplementary material for: Large Animal Emergency Relief Services—A Model for University Engagement With Private Practitioners and Development of Practice Readiness for Veterinary Students
Source: Front Vet Sci. 2020 Jul 17;7:403. doi: 10.3389/fvets.2020.00403 (PMC7396559; doi:10.3389/fvets.2020.00403)
Supplement: Supplement 1 — Example personalized introductory letter to an area veterinarian announcing the new emergency service provided by the University of Tennessee College of Veterinary Medicine. [file Data_Sheet_1.pdf]

November 28, 2013

Dear _______________,

My name is -------. I have recently relocated to your area from North Carolina. I have been in equine practice since graduation from the University of Tennessee in 2001. After having my first child ten years ago, I began doing relief work as an equine emergency practitioner and thoroughly enjoyed it. Recognizing the need for competent, enthusiastic emergency care for large animals is one of the initial reasons I even studied veterinary medicine!

In recent years, many small animal practitioners have been able to utilize the much appreciated services of after-hours clinics. Unfortunately, large animal veterinarians have never had the luxury of 'turning their phones over to an emergency clinic.' I hope to provide this service in East Tennessee.

For the past five years, I have been covering large animal emergencies in western North Carolina for seven surrounding practices. Now having moved back to Tennessee, I plan to offer this same service in your area. To do this, I have partnered with the University of Tennessee College of Veterinary Medicine and plan to practice out of their trucks and with their students. UTCVM sees this as an excellent opportunity to provide a community service to area veterinarians, as well as, to help develop 'practice-ready' graduates.

My protocol is to treat or stabilize patients until they can be maintained by their regular provider. All calls received will ring directly to my cell phone and will be answered by me personally. There will be no operator or answering service involved on our end. I will not be providing any routine services (e.g.: vaccines, teeth floating, coggins, etc.), therefore eliminating any "threat" to the regular veterinarian. Your clients will be instructed to follow-up with their regular veterinarian for any continued care needed. This is strictly an emergency service.

Clients will be billed by the UTCVM, thus eliminating any paperwork or billing hassles on your end. Please understand that there is no fee to you for our service! If you would like copies of your patient's records and treatments received, simply contact the UTCVM Equine Hospital.

We are currently working on an official launch date and a schedule of availability. We will be contacting you soon with these details. Please feel free to contact me if you have any questions regarding this new service available to you to alleviate some of your emergency load. My cell phone number is: #######. You may also e-mail me at ---@utk.edu. I would appreciate any feedback you could provide me as well. I look forward to meeting you and hopefully establishing a working relationship with you.

Sincerely,

Meggan T. Graves, DVM
