# Supplementary material for: Large Animal Emergency Relief Services—A Model for University Engagement With Private Practitioners and Development of Practice Readiness for Veterinary Students
Source: Front Vet Sci. 2020 Jul 17;7:403. doi: 10.3389/fvets.2020.00403 (PMC7396559; doi:10.3389/fvets.2020.00403)
Supplement: Supplement 2 — Percentage change of revenue generated for fiscal year 2014 to 2019 from an after-hours, field-based, emergency relief service. [file Data_Sheet_2.pdf]

	**Year 1**	**Year 2**	**Year 3**	**Year 4**	**Year 5**
**Equine Field Service Emergency**	72%	-16%	5%	-7%	21%
**Food Animal Field Service Emergency**	98%	-1%	24%	3%	-1%
**Total Field Service Emergency**	85%	-9%	15%	-2%	10%

Supplement 2: Percentage change of revenue generated since the formation of an after- hours, field-based, emergency relief service.
